# Nanomechanical DNA Origami pH Sensors

**DOI:** 10.3390/s141019329

**Published:** 2014-10-16

**Authors:** Akinori Kuzuya, Ryosuke Watanabe, Yusei Yamanaka, Takuya Tamaki, Masafumi Kaino, Yuichi Ohya

**Affiliations:** 1 Department of Chemistry and Materials Engineering, Kansai University, 3-3-35 Yamate, Suita, Osaka 564-8680, Japan; E-Mails: k947410@kansai-u.ac.jp (R.W.); y7yamanaka@gmail.com (Y.Y.); tamaki.takuya@mw.nkchemical.co.jp (T.T.); k460583@kansai-u.ac.jp (M.K.); 2 PRESTO, Japan Science and Technology Agency, 4-1-8 Honcho, Kawaguchi, Saitama 332-0012, Japan

**Keywords:** DNA Origami, DNA Nanotechnology, Proton Detection, pH Sensors, i-motif, AFM, nanomechanical devices, Single-Molecule Sensors

## Abstract

Single-molecule pH sensors have been developed by utilizing molecular imaging of pH-responsive shape transition of nanomechanical DNA origami devices with atomic force microscopy (AFM). Short DNA fragments that can form i-motifs were introduced to nanomechanical DNA origami devices with pliers-like shape (DNA Origami Pliers), which consist of two levers of 170-nm long and 20-nm wide connected at a Holliday-junction fulcrum. DNA Origami Pliers can be observed as in three distinct forms; cross, antiparallel and parallel forms, and cross form is the dominant species when no additional interaction is introduced to DNA Origami Pliers. Introduction of nine pairs of 12-mer sequence (5′-AACCCCAACCCC-3′), which dimerize into i-motif quadruplexes upon protonation of cytosine, drives transition of DNA Origami Pliers from open cross form into closed parallel form under acidic conditions. Such pH-dependent transition was clearly imaged on mica in molecular resolution by AFM, showing potential application of the system to single-molecular pH sensors.

## 1. Introduction

Structural DNA nanotechnology [1], based on the programmed assembly of branched DNA helices, has provided various functional nanomechanical devices. DNA devices such as molecular beacons, DNA tweezers, and DNA walkers have been constructed to date [2–12]. A DNA nanomachine-based pH sensor is one of the most successful achievements in the field that enabled spatial and temporal pH mapping inside living cells [13–15]. DNA origami [16,17], in which long single-stranded DNA is folded into a designed nanostructure with the aid of many short staple strands, is a powerful new tool in structural DNA nanotechnology that provides robust and precise nanostructures in both 2D and 3D that are even visible with atomic force microscopy (AFM) or electron microscopy (EM). Despite these properties, only limited studies on DNA origami as a building material for nanomechanical DNA devices have been published to date [18].

We have recently developed a nanomechanical DNA origami device (DNA Origami Pliers and DNA Origami Forceps) by joining two stick-like components of 170-nm long (levers of the pliers) at a fulcrum. DNA Origami Pliers thus can take three conformations: open cross form in which the two levers are not tied to each other and are in X-shape connected at the fulcrum, parallel and antiparallel closed forms in which two levers are aligned horizontally by the addition of the second or more bridges between the levers [19–21]. We applied them to construct detection systems for biomolecules, which are usually too small to be individually imaged, in single-molecular resolution by observing the structure switching of DNA Origami Pliers under AFM. A variety of bio-related targets from inorganic metal ions of a few tens of atomic weight to organic antibodies with molecular weights of up to 150 kDa triggered the structure switching by specific interactions between the targets and the ligands attached to the nanomechanical DNA origami devices [19].

In this study, we intended to expand the range of the target for DNA Origami Pliers, and chose proton as a new target for the detection system. We utilized i-motif quadruplex formation as the trigger for shape transition of DNA Origami Pliers. We have successfully and clearly observed pH-dependent closing of DNA Origami Pliers bearing nine pairs of i-motif-forming 12-mer strands by using AFM.

## 2. Experimental Section

### 2.1. Materials

Staple strands were purchased from Integrated DNA Technologies (Coralville, IA, USA) under standard desalting grade and used without further purification. M13mp18 ssDNA (Takara, Japan) was used for the DNA origami scaffold.

### 2.2. Preparation of DNA Origami Pliers

Formation of DNA Origami Pliers in open cross form (initial state) was performed with M13mp18 ssDNA (4 nM) and staple strands (20 nM for each strand) in a solution containing Tris (40 mM), acetic acid (20 mM), EDTA (10 mM), and magnesium acetate (12.5 mM, 1X TAE/Mg^2+^ buffer, 50 μL). This mixture was cooled from 90 °C to 25 °C at a rate of −1.0 °C/min using a PCR thermal cycler to anneal the strands.

### 2.3. pH-Dependent Form Transition and AFM Imaging

Structure switching of DNA Origami Pliers was done by adding nine equivalents of appropriate buffer to the solution of DNA Origami Pliers followed by ultrafiltration. For example, 4 nM parallel DNA origami pliers annealed in 1X TAE/Mg^2+^ buffer (50 μL) was added 450 μL of 40 mM HEPES (pH = 7.0 or 6.0) containing 12.5 mM Mg^2+^, and the mixture was applied to an Amicon Ultra 50K ultracentrifuge filter (Millipore, Billerica, MA, USA). After 10 min centrifugation at 7800 rpm at 15 °C, the remainder was re-suspended in HEPES buffer and was subjected to AFM imaging. For imaging at pH 5.6, MES buffer was used instead of HEPES.

AFM imaging of DNA Origami Pliers was performed on a Multimode 8/Nanoscope V system (Bruker AXS). The solution of DNA Origami Pliers (1 μL) was deposited on a freshly cleaved mica substrate, additional buffered solution (40 μL) was added, and the imaging was performed using the fluid PeakForce Tapping mode with a BL-AC40TS tip (Olympus, Japan). DNA origami devices in images were counted as open cross form when both of the ends were clearly separated AND the levers around the fulcrum were clearly not laid in parallel. They were counted as parallel closed form when at least one of the ends was clearly identified to be in head-to-head (the end of a lever close to the concavity) or tail-to-tail (the opposite end of the lever) contact, or into antiparallel form when head-to-tail contact was clearly observed for at least one of the ends. Motifs not in any of the above conditions were counted as unclear motifs. The classification was confirmed by at least three individuals.

## 3. Results and Discussion

**Figure 1.**
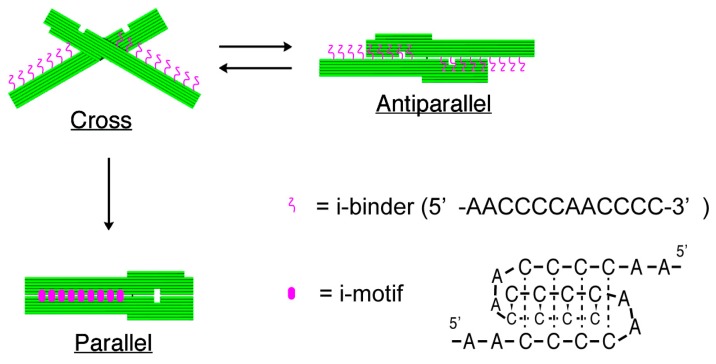
Schematic illustration of the present system. Under acidic conditions, nine pairs of 12-mer i-binders (5′-AACCCCAACCCC-3′) attached to the levers of DNA Origami Pliers form i-motif quadruplexes by protonation of the cytidines.

The structures of nanomechanical DNA origami devices, “DNA Origami Pliers”, and the binders attached to them are shown in Figure 1 (see Figure S1 for detailed design with DNA sequences). DNA Origami Pliers consist of two *ca.* 170-nm long lever domains, which are made of six parallel DNA helices of M13 scaffold assembled in a raft-like planar structure. These levers are joined together at a fulcrum via two phosphodiester linkages in the M13 scaffold so that an immobile Holliday junction is formed between them. Such DNA four-way junction is known to be in a right-handed, antiparallel stacked X-structure with a small angle of 60° in the presence of Mg^2+^, and thus DNA Origami Pliers are usually observed as X-shaped open form (cross form) in AFM images on mica.

To introduce pH responsive function to DNA Origami Pliers, nine pairs in total of 12-mer DNA sequence (5′-AACCCCAACCCC-3′, i-binders, drawn in magenta wavy lines) were attached to the lever portions. The 3′-end of staple strands placed in the lever portions were extended with the i-binders so that the binders are placed in the potential positions for DNA crossovers, which usually appear every 32 nucleotides in standard DNA origami design (see Table S1 for the sequences of the staples with i-binders). These i-binders dimerize into a quadruplex called “i-motif” as a result of formation of C-C^+^ unusual basepairs upon protonation of cytosines under acidic conditions [13,14].

**Figure 2.**
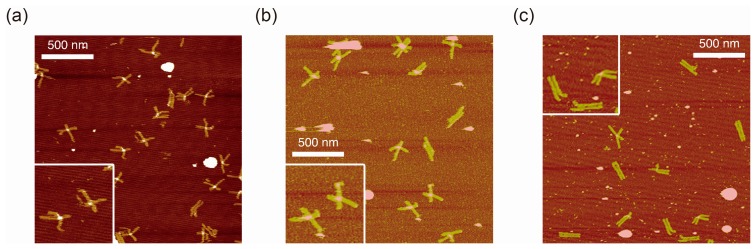
Atomic force microscopy (AFM) images of DNA Origami Pliers deposited on mica at pH 8.2 (**a**); pH 7.0 (**b**); and pH 5.6 (**c**). Insets: 150% magnified view of typical motifs.

**Figure 3.**
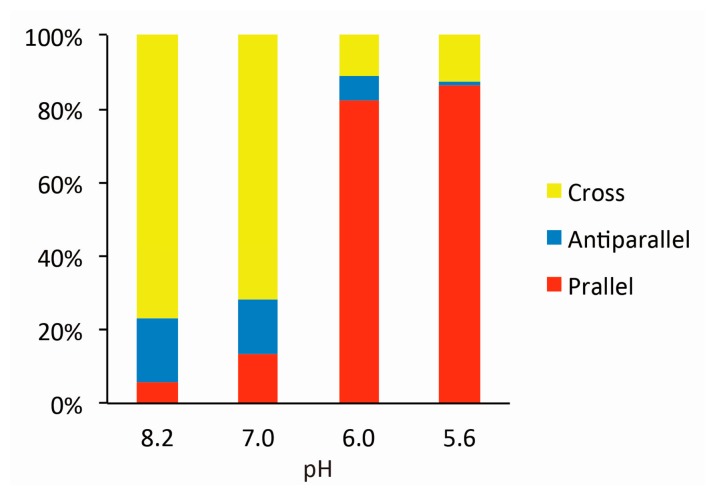
Distribution of three forms of DNA Origami Pliers in AFM images at various pH.

Figure 2 shows typical AFM images of i-binder-modified DNA Origami Pliers deposited on mica in 1X TAE/Mg^2+^ buffer at pH 8.2 (a), in HEPES/Mg^2+^ buffer at pH 7.0 (b), or in MES/Mg^2+^ buffer at pH 5.6 (c). At pH 8.2, which is a standard condition to assemble and handle DNA nanostructures including DNA origami, most of the motifs observed in the images were in open cross form, and motifs in parallel closed form were few. The distributions of each forms estimated by counting the number of motifs in the AFM images (Table S2) are shown in Figure 3. Nearly 80% of DNA Origami Pliers was in open cross form (yellow bar), and the yields of antiparallel and parallel closed forms were 17% and 6%, respectively. These numbers are in quite good accordance with our previous results [19,21]. Similar distribution, but with slight increase of parallel closed form in exchange for open cross form, was obtained when the pH was lowered to 7.0 (Figure 2b). Landscape of AFM images under acidic conditions significantly altered as shown in Figure 2c. Here, most of DNA Origami Pliers took parallel closed form in MES/Mg^2+^ buffer at pH 5.6. Almost 87% of observed motifs were in parallel closed form, and the yield of open cross form was only 12%. This large difference is solely caused by pH difference, not by the alteration of buffer compound; since parallel closed form was also dominant in HEPES buffered solution at pH 6.0 (Figure 3). It is obvious that the threshold of the closing of DNA Origami Pliers exists between pH 7 and 6.

Introduction of four pairs of i-binders, which had been enough to achieve sufficient closing of DNA Origami Pliers when G-quartet-forming binders were introduced for K^+^ detection, in contrast, gave only 34% parallel closed forms (Figure S2). This may represent relatively week interaction between the binders, but still significant dependence of the yield of parallel forms on the number of i-binders clearly shows that the closing of DNA Origami Pliers is indeed driven by the formation of i-motifs between the binders.

As expected, reversal of selectively closed DNA Origami Pliers in acidic conditions into open cross form was also successful by re-exchanging the buffer to 1X TAE/Mg^2+^ at pH 8.2 again (Figure S2).

## 4. Conclusions

We have successfully controlled the structure of nanomechanical DNA origami devices by pH of the solution. The i-binder-modified DNA Origami Pliers were selectively and efficiently closed into parallel form by acidifying the solution to pH 6. AFM imaging of DNA origami (∼1 nM) on mica can be achieved with fairly small amount of sample solution (∼0.1 μL) that the sensitivity of the present system is not less high than conventional pH meters. Functionalization of DNA origami is not limited to only one ligand but multiple kinds of ligands can be freely attached to the levers of DNA Origami Pliers in any combinations. Moreover, the shape of DNA origami devices can be also flexibly altered for precise discrimination between multiple DNA origami devices targeting different biomolecules. Structure switching of DNA Origami Pliers can be monitored not only by AFM imaging but also by agarose gel electrophoresis [20], or by fluorescent spectroscopy even in real-time. These advantages should enable highly sophisticated multiplex detection systems of biomolecules in single-molecule resolution.

## Supplementary Material
