# Supplementary material for: Nanomechanical DNA Origami pH Sensors
Source: Sensors (Basel). 2014 Oct 16;14(10):19329–35. doi: 10.3390/s141019329 (PMC4239864; doi:10.3390/s141019329)
Supplement: Supplementary file 1 [file sensors-14-19329-s001.pdf]

OPEN ACCESS

sensors

ISSN 1424-8220

www.mdpi.com/journal/sensors

Supplementary Information

Nanomechanical DNA Origami pH Sensors. *Sensors* 2014, *14*, 19329-19335

Akinori Kuzuya [1,2,*], Ryosuke Watanabe [1], Yusei Yamanaka [1], Takuya Tamaki [1], Masafumi Kaino [1] and Yuichi Ohya [1,*]

[1] Department of Chemistry and Materials Engineering, Kansai University, 3-3-35 Yamate, Suita, Osaka 564-8680, Japan; E-Mails: k947410@kansai-u.ac.jp (R.W.); va80235@kansai-u.ac.jp (Y.Y.); k450228@kansai-u.ac.jp (T.T.); k460583@kansai-u.ac.jp (M.K.)

[2] PRESTO, Japan Science and Technology Agency, 4-1-8 Honcho, Kawaguchi, Saitama 332-0012, Japan

* Authors to whom correspondence should be addressed; E-Mails: kuzuya@kansai-u.ac.jp (A.K.); yohya@kansai-u.ac.jp (Y.O.); Tel./Fax: +81-6-6368-0829 (A.K.); +81-6-6368-0818 (Y.O.).

Table S1. Sequences of the staples bearing i-binders and those used in combination with them.

Pliers24shortisw	TGTAGCATCCAGACGTTAGTAAATTTTTAACCCCAACCCC
Pliers33shortisw	TGAGTTTCATTTTGCTAAACAACTTTTTAACCCCAACCCC
Pliers53shortisw	CAGGGATATAGAAAGGAACAACTATTTTAACCCCAACCCC
Pliers58shortisw	CACCCTCAACGTTGAAAATCTCCATTTTAACCCCAACCCC
Pliers62shortisw	AGGAGGTTAGGAGCCTTTAATTGTTTTTAACCCCAACCCC
Pliers66shortisw	GTATAGCCGCTTTCGAGGTGAATTTTTTAACCCCAACCCC
Pliers70shortisw	GCGGATAAACCGATAGTTGCGCCGTTTTAACCCCAACCCC
Pliers74shortisw	ATTAGGATTCGCCCACGCATAACCTTTTAACCCCAACCCC
Pliers78sisw	GTATTAAGGAGGCTTGCAGGGAGTTTTTAACCCCAACCCC
Pliers166sisw	GTCGAGGTAGAGATAGAACCCTTCTTTTAACCCCAACCCC
Pliers170shortisw	CTAAAGGGCGACCAGTAATAAAAGTTTTAACCCCAACCCC
Pliers174shortisw	GGAAAGCCGATTATTTACATTGGCTTTTAACCCCAACCCC
Pliers178shortisw	GAAGAAAGACCTACATTTTGACGCTTTTAACCCCAACCCC
Pliers182shortisw	TGGCAAGTGCCATTGCAACAGGAATTTTAACCCCAACCCC
Pliers186shortisw	ACCACACCTGCTGGTAATATCCAGTTTTAACCCCAACCCC
Pliers190shortisw	TATGGTTGTAACATCACTTGCCTGTTTTAACCCCAACCCC
Pliers206shortisw	TAGAATCAACGCAAATTAACCGTTTTTTAACCCCAACCCC
Pliers222shortisw	ATTAAAGGGTGAGGCCACCGAGTATTTTAACCCCAACCCC
Pliers13slong	AAATCACCAGTAGCAGGCATTTTCGGTCATGTAACAC
Pliers23long	GAATTTTCTGTATGGGGTCACCAGTACAAACTGTAGCGCG
Pliers35+41	TTCAACAGTTTCAGCGGAGTGAGAAGCAAGCCCAATAGGAA
Pliers54long	AAGGAATTGCGAATAATAATTTTTTCGAACCGCCACCCTCAG
Pliers57long	AAAAAAAGGCTCCAAATAGTACCGCCACCCTCGCCACCCT
Pliers61long	ATCGGTTTATCAGCTTCGGAATAGGTGTATCACACCCTCA
Pliers181long	AAACGCTCATGGAAATCGAAAGGAGCGGGCGCCACCAGTG
Pliers185long	AACAATATTACCGCCAGTAGCGGTCACGCTGCTGCGTATT
Pliers189long	AGTAGAAGAACTCAAACTATCGGCCTCGCCGCGCTTAATGCG
Pliers204+205	GTAGCAATACTTCTTTGATTAGTAACTTTGACGAGCACGTA
Pliers210long	ACCGAGCTCGAATTCTCACATTAATTGCGTTCCTCGTT
Pliers221long	AAAGAGTCTGTCCATCGAGCGGGAGCTAAACATAATGAGT

Figure S1. Detailed structure of DNA Origami Pliers bearing i-binders. Positions of i-binder attachment are indicated by extensions of the staples. For a list of staples, see [1].

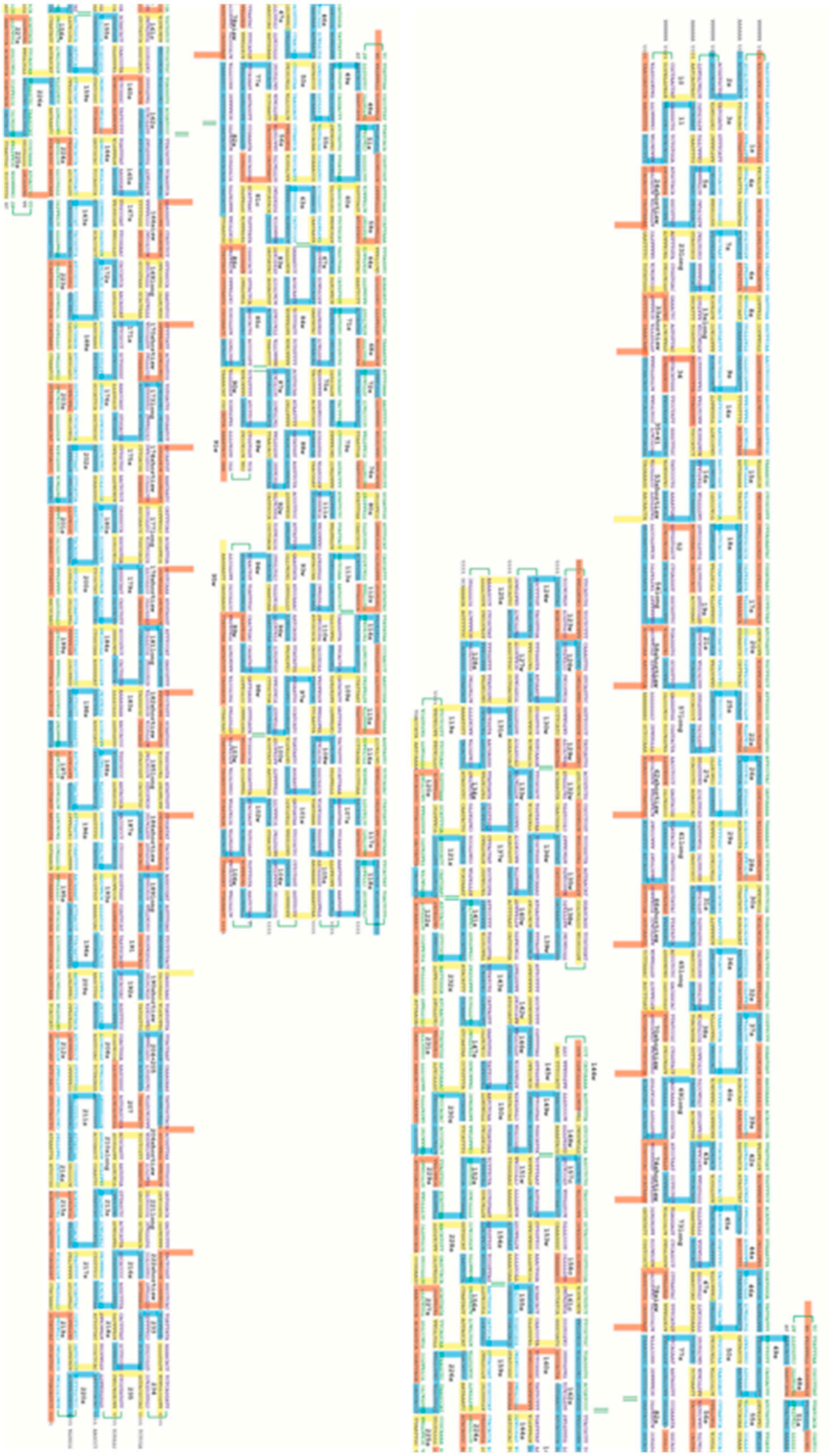

Table S2. Counted numbers of the motifs in AFM images.

pH	8.2	7	6	5.6
cross	121	138	5	12
antiparallel	27	28	3	1
parallel	9	26	37	84
sum	157	192	45	97

Figure S2. Reversible pH-dependent structure switching of DNA Origami Pliers with four pairs of i-binders.

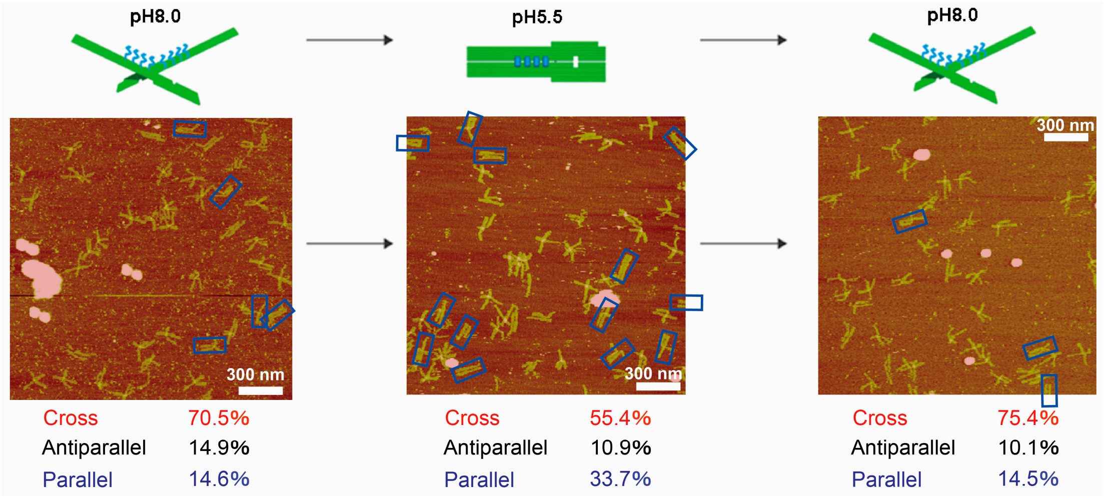

Reference

1. Kuzuya, A.; Sakai, Y.; Yamazaki, T.; Xu, Y.; Komiyama, M. Nanomechanical DNA Origami “Single-Molecule Beacons” Directly Imaged by Atomic Force Microscopy. *Nat. Commun*. **2011**, *2*, doi:10.1038/ncomms1452.

© 2014 by the authors; licensee MDPI, Basel, Switzerland. This article is an open access article distributed under the terms and conditions of the Creative Commons Attribution license (http://creativecommons.org/licenses/by/4.0/).
